# ﻿Two new species of *Columnea* (Gesneriaceae) from the Colombian Andes

**DOI:** 10.3897/phytokeys.261.160135

**Published:** 2025-08-22

**Authors:** John L. Clark, David Lozano-Cifuentes, Jorge Ríos-Cervera, Laura Clavijo, Sofía Ballesteros

**Affiliations:** 1 Marie Selby Botanical Gardens, 1534 Mound St., Sarasota, FL 34236, USA Marie Selby Botanical Gardens Sarasota United States of America; 2 Parques Nacionales Naturales de Colombia – Dirección Territorial Orinoquía, Carrera 39 #26C-47, Villavicencio, Meta, Colombia Parques Nacionales Naturales de Colombia Meta Colombia; 3 Programa de Ingeniería Forestal, Facultad de Ingeniería Forestal, Universidad del Tolima, Calle 42 #1B-1, Ibagué, Colombia Universidad del Tolima Ibagué Colombia; 4 Universidad Nacional de Colombia–Sede Bogotá, Facultad de Ciencias, Instituto de Ciencias Naturales, Bogotá, D.C., 111321, Colombia Universidad Nacional de Colombia Bogotá Colombia

**Keywords:** Andes, Columneinae, Gesnerieae, Gesnerioideae, taxonomy

## Abstract

Two new species of *Columnea* are described based on recent field expeditions and herbarium research. These species are unusual within *Columnea* for exhibiting a dorsiventral epiphytic habit typical of many northern Andean species, but they lack conspicuous floral bracts. *Columnea combeimae* Lozano-Cif., J.E.Ríos & J.L.Clark, **sp. nov.** is distinguished by uniformly green leaves, prominent extrafloral nectary glands on the pedicels, and a hirsute indumentum of purple trichomes. *Columnea rubropilosa* J.L.Clark & Clavijo, **sp. nov.** is characterized by a conic purple berry and a densely pilose indumentum of red trichomes on both vegetative and reproductive structures. Both species are endemic to the Colombian Andes.

## ﻿Introduction

The flowering plant family Gesneriaceae, with more than 3,900 species and 150+ genera (GRC 2025), is in the order Lamiales. The family is divided into three subfamilies and seven tribes (Weber et al. 2013, 2020), which represent strongly supported monophyletic lineages based on DNA sequence data (Ogutcen et al. 2021). The majority of the neotropical members are in the subfamily Gesnerioideae and are represented by 1,200+ species and 77 genera (Clark et al. 2020; GRC 2025). The genus *Columnea* L. is classified in the tribe Gesnerieae and the subtribe Columneinae (Weber et al. 2013, 2020). *Columnea* is represented by a broad range of habits ranging from epiphytes with dorsiventral shoots to pendent vines with elongate shoots. Flower shapes range from hypocyrtoid, campanulate, or deeply bilabiate. The single defining morphological synapomorphy that distinguishes *Columnea* is an indehiscent berry fruit, in contrast to the more common bivalved fleshy capsule found in most members of the subtribe Columneinae.

In Colombia, the genus *Columnea* is especially diverse and abundant, with at least 80 species documented by the 1990s (Kvist and Skog 1993; Kvist et al. 1998). Subsequent research has raised the number of described species in Colombia to approximately 106 (GRC 2025) underscoring the importance of continued taxonomic and collections-based research. Recent exploratory fieldwork and herbarium research on the Colombian flora have revealed two undescribed species that share a dorsiventral habit with inconspicuous floral bracts. The new species *Columnea combeimae* Lozano-Cif., J.E.Ríos & J.L.Clark is differentiated from *Columnea fuscihirta* L.P.Kvist & L.E.Skog. The new species *Columnea rubropilosa* J.L.Clark & Clavijo is differentiated from *C. purpurata* Hanst. Our ongoing investigations have led to the formal description of these two new species, which are documented here with detailed morphological descriptions, ecological notes, preliminary conservation assessments, and field images.

The monophyly of *Columnea* is strongly supported by molecular phylogenetic studies (Clark et al. 2006; Smith et al. 2013; Schulte et al. 2014). Subgeneric classifications and traditionally recognized subgenera are mostly artificially defined and lack support from recent phylogenetic studies (Smith and Carroll 1997; Smith 2000; Clark and Zimmer 2003; Clark et al. 2012; Smith et al. 2013; Schulte et al. 2014). Thus, this study refrains from assigning the two described *Columnea* species here to a subgeneric or traditionally classified group.

## ﻿Materials and methods

Specimens were collected in the field and subsequently pressed and dried. Specimens are currently deposited at the National Herbarium of Colombia (COL) and the herbarium at the Universidad Surcolombiana (SURCO). Additional specimens will be distributed to Marie Selby Botanical Gardens (SEL), Bogotá Botanical Garden Jose Celestino Mutis (JBB), Missouri Botanical Garden (MO), New York Botanical Garden (NY), and US National Museum of Natural History (US). Photographs were taken of live specimens in the field using a Nikon D5300 with a Nikon AF-S FX Nikkor 50 mm, f/1.8D lens. Morphological observations and measurements were made from live collections, alcohol-preserved material, and digital images using the software program *ImageJ* (Schneider et al. 2012).

The extinction risk was assessed following the IUCN Red List Categories and Criteria (IUCN 2012) and updated criteria in the IUCN Standards and Petitions Committee (2024). Field observations and collection sites from fieldwork were used to evaluate the IUCN category. The extent of occurrence (EOO) and area of occupancy (AOO) were calculated using the software program *GeoCAT* (Bachman et al. 2011) with the default setting of 2 km, which is a 4 km^2^ grid cell. Observations from iNaturalist were also considered when calculating the AOO and EOO.

## ﻿Taxonomic treatment

### ﻿ Columnea combeimae

Taxon classificationPlantaeLamialesGesneriaceae

Lozano-Cif., J.E.Ríos & J.L.Clark sp. nov.

08B6F479-12BA-56B5-B7DB-6AEE4967EF40

urn:lsid:ipni.org:names:77367930-1

Fig. 1

#### Type.

**Colombia** • Tolima: Ibagué, vereda Ancón, Tesorito, ruta de las tres cascadas, 4°28'49.19"N, 75°13'27.54"W, 1900 m, 21 Mar 2024 (fl.), *D. Lozano-Cifuentes, J. Ríos & N. Romero 554* (holotype: SURCO! [accession 016300]; isotypes: SEL, JAUM).

#### Diagnosis.

*Columnea combeimae* and *C. fuscihirta* share a dorsiventral epiphytic habit, elongate tubular yellow corollas, and conspicuous flowers lacking showy floral bracts. *Columnea combeimae* differs in having a uniformly green abaxial leaf surface (vs. abaxial surface with red apices in *C. fuscihirta*), a glabrescent region at the base of the shoots (vs. shoots uniformly hirsute), and prominent nectary glands at the apex of the pedicel (vs. nectaries absent on the pedicel).

#### Description.

Facultative epiphytic herbs with dorsiventral shoots; stems terete in cross-section, 1.7–2.2 cm in diameter, glabrescent at the base and densely hirsute with dark purple trichomes toward the apex; internodes 0.8–3.6 cm long, leaf scars flush with the stem. ***Leaves*** opposite, anisophyllous, larger blade broadly lanceolate to oblong, 7.5–25 cm long, 3.5–7.0 cm wide, apex acuminate, base oblique, lateral veins 6–8 per side, adaxially uniformly dark green, abaxial surface uniformly light green with prominent purple midvein for lower third and otherwise green, abaxial leaf surface uniformly pilose to hirsute with multicellular purplish-colored trichomes, denser on veins, margin crenulate to serrulate; petioles (0.8–)1.0–1.8(–2.2) cm long, pilose with multicellular purplish-colored trichomes; smaller blade 0.7–1.5 cm long, 0.4–0.7 cm wide, lateral veins 1–3 per side, petiole 0.6–0.9 mm, otherwise similar to large blade. ***Inflorescence*** reduced to 1–3 axillary flowers; bracts inconspicuous, ovate, purple, 4–5 mm long, 4.2–4.6 mm wide, slightly acuminate apex. ***Flowers*** with pedicels 3–6 mm long, light green, densely pilose with multicellular gold-colored hispid indumentum. ***Calyx*** lobes green with purplish apices, 1.7–2.2 cm long, 0.7–1.1 cm wide at base, oblong, apex acuminate and reflexed; outer surface densely pilose to hirsute with multicellular purplish trichomes; inner surface glabrous; margin with at least two large laciniate projections per side. ***Corolla*** 3.2–4.1 cm long, 0.8–1.0 cm wide at widest point and 0.6–0.7 cm at base, tubular, lobes elliptic, tube 2.8–3.3 cm long, corolla lobes 0.4–0.5 mm long, rounded, slightly ampliated on lower surface, gibbous at base, uniformly yellow, outer surface densely pilose with multicellular gold-colored trichomes, inner surface of corolla tube with glandular trichomes. ***Androecium*** of 4 didynamous stamens, filaments glabrous, ca. 2.7–3.0 cm long, connate at base for 0.3–0.4 cm and adnate to corolla, anthers ca. 2.5–2.7 mm long, 1.9–2.1 mm wide, quadrangular. ***Gynoecium*** with nectary comprised of a single-lobed dorsal and two smaller lateral glands, ovary ca. 3.0–4.5 mm long, globose, indument pilose; style 2.0–2.6 cm long, glabrescent, stigma stomatomorphic. ***Fruits*** not observed.

#### Additional specimens examined.

**Colombia – Tolima.** • Ibagué: ruta sur al nevado del Tolima, entre lajas y tierra de gigantes, 4°22'23"N, 75°13'58.81"W, 3200 m, 21 Mar 2024 (fl.), *D. Lozano-Cifuentes & M. Rincón 95* (JBB [accession: 41534]); • Líbano, 4°54'10"N, 75°6'10"W, 2340 m, 7 Oct 2022 (fl.), *J. Betancur et al. 23775* (COL [accession: 628308]); • Murillo, vereda el agrado, km 7.5 vía Murillo, hostal Camino Viejo, 4.90220°N, 75.10281°W, 2169 m, 9 Oct 2022 (fl.), *J. Aguirre-Santoro et al. 5059* (COL [accession: 626998]).

#### Phenology.

Plants were documented in flower during January to March and October to December.

#### Etymology.

The specific epithet refers to the Combeima Canyon (Cañón de Combeima), the type locality where the species was discovered.

#### Distribution and preliminary IUCN red list assessment.

*Columnea combeimae* is endemic to Colombia and known from only three collections in high Andean forests of the Cordillera Central, within the department of Tolima and the municipalities of Ibagué and Libano, at elevations between 2000 and 3500 m. Its extent of occurrence (EOO) is estimated at 35.8 km^2^, and its area of occupancy (AOO) is estimated at 12 km^2^. One locality occurs in a recently disturbed area of Parque Nacional Natural Los Nevados, while the other two are in forested areas with no formal protection.

The species is threatened by ongoing habitat loss and fragmentation resulting from deforestation, the expansion of agriculture (especially coffee plantations), livestock, and urbanization. These threats were recently observed by the authors and have led to a recent decline in the quality and extent of suitable habitat (subcriteria B2b(i, ii, iii) and B1b(i,ii,iii)). The EOO is within the threshold of Critically Endangered (<100 km^2^) and the AOO is within the threshold of Endangered (<50 km^2^). The number of known locations is three, which is within the threshold of Endangered (number of known locations < 5). *Columnea combeimae* is preliminary assessed as Endangered (EN) under IUCN Red List criteria B1ab(i,ii,iii) +2ab(i,ii,iii).

#### Comments.

**Figure 1.**
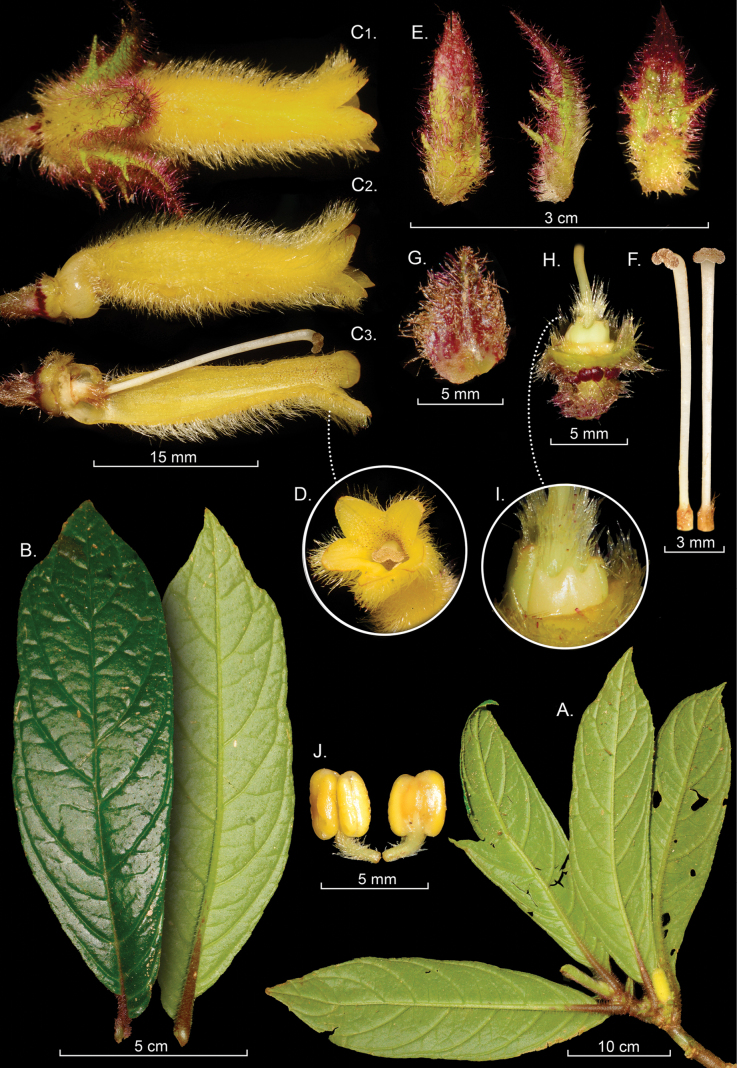
*
Columnea combeimae* J.Lozano-Cif., J.E.Ríos & J.L.Clark. A. Habit; B. Abaxial and adaxial leaf surfaces; C. Lateral views of flower; D. Front view of corolla; E. Calyx lobes; F. Stigma and style; G. Floral bract; H, I. Gynoecium featuring nectaries; J. Androecium (A–I. from *D. Lozano-Cifuentes et al. 554*). Photos by David Lozano-Cifuentes.

**Figure 2.**
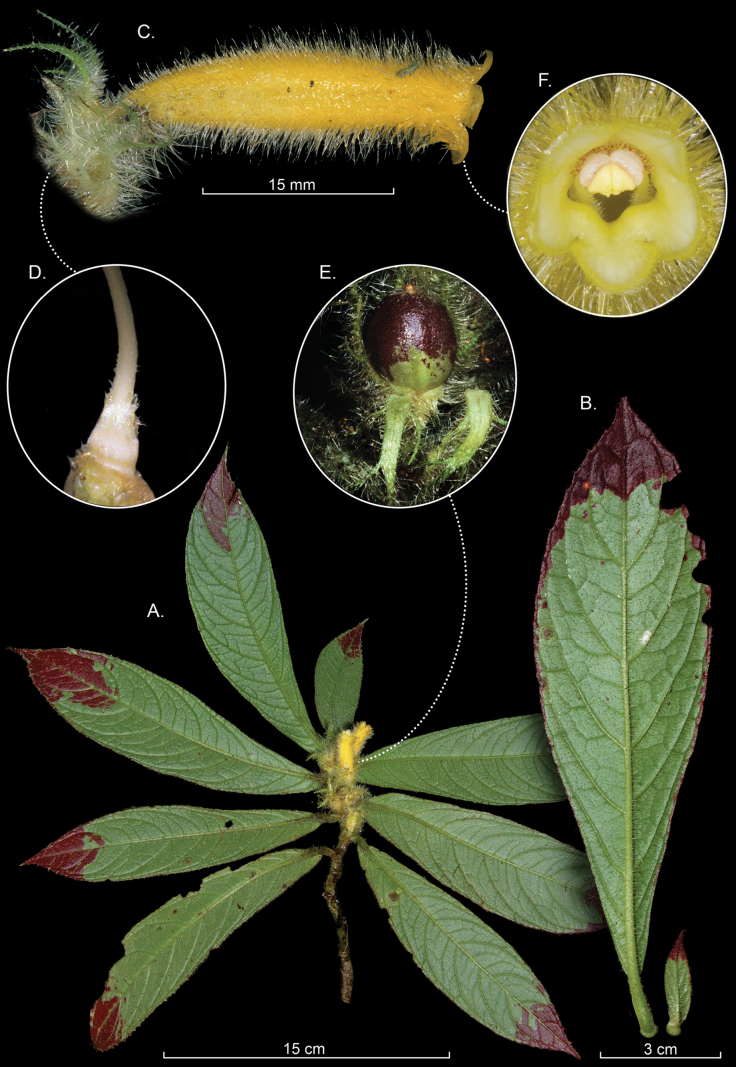
*
Columnea fuscihirta* L.P.Kvist & L.E.Skog. A. Habit; B. Abaxial leaf surface; C. Lateral view of flower; D. Gynoecium; E. Immature fruit; F. Front view of corolla (A, B. from *Oscar Marin s.n*. C–F. from *J.L. Clark et al. 15808*). Photos A, B. by Oscar Marin and C–F. by John L. Clark.

*Columnea combeimae* (Fig. 1) and *C. fuscihirta* (Fig. 2) are similar in having uniformly bright yellow tubular corollas (Figs 1C, 2C) and inconspicuous or highly reduced bracts. In contrast, most species of *Columnea* have persistent brightly colored bracts that cover the calyx lobes and often the lower portion of the corolla tube. Both *C. combeimae* and *C. fuscihirta* share a dorsiventral epiphytic habit and pairs of anisophyllous leaves (Figs 1B, 2A).

*Columnea combeimae* is distinguished from *C. fuscihirta* by the presence of a hirsute indumentum of purple trichomes near the shoot apex and glabrescent basally. In contrast, *C. fuscihirta* has a uniformly golden-yellow indumentum that is consistently hirsute at both the base and apex of the shoots. The abaxial leaf surface of *C. fuscihirta* is green with red apices (Fig. 2B), whereas in *C. combeimae* it is uniformly green (Fig. 1A, B). Additional diagnostic features of *C. combeimae* include a purple midvein on the lower surface of the leaf (vs. uniformly green in *C. fuscihirta*) and prominent extrafloral nectary glands on the upper portion of the pedicel (Fig. 1H), which are absent in *C. fuscihirta*.

### ﻿ Columnea rubropilosa

Taxon classificationPlantaeLamialesGesneriaceae

J.L.Clark & Clavijo sp. nov.

54F91360-2046-5A18-9C32-67C3EEC3BE0D

urn:lsid:ipni.org:names:77367932-1

Fig. 3

#### Type.

**Colombia** • Antioquia: municipio Jardin, vereda Macayas, eastern slopes of the Cordillera Occidental, Reserva biológica El Centello (Jardin Botánico Medellín), camino la Risaralda, 2443 m, 5°30'8.69"N, 75°51'0.59"W, 8 Aug 2024 (fl. & fr.), *J.L. Clark, Á. Idárraga, S. Ballesteros, N. Salinas & L. Clavijo 19235* (holotype: COL!; isotypes: SEL, MO, NY).

#### Diagnosis.

*Columnea rubropilosa* and *C. purpurata* share a dorsiventral epiphytic habit, elongate tubular corollas, and fimbriate calyx margins. *Columnea rubropilosa* differs by a conic purple berry (vs. globose orange berry in *C. purpurata*) and a pilose indumentum of red trichomes on vegetative and reproductive structures (vs. a pilose indumentum of white trichomes on vegetative and reproductive structures in *C. purpurata*).

#### Description.

Facultative epiphytic herbs with dorsiventral shoots; stems scandent, branched, up to 50 cm long, terete in cross-section, 1.9–5.2 mm diameter, herbaceous to subwoody, maroon, surface rugose, uniformly pilose at the base and densely pilose toward the apex, trichomes dark red-colored, branched, up to 3.0–3.5 mm long; internodes 1.7–4.0 cm long. ***Leaves*** opposite, anisophyllous; larger blade with petiole 0.9–1.8 cm long, terete, densely pilose, covered by branched, multicellular, and dark red trichomes, up to 3.0–3.5 mm long; blade broadly lanceolate to oblong, 8.0–16.0 × 2.0–4.0 cm, rigid and nearly sclerophyllous, dark green adaxially, light green abaxially with the margin, main, and lateral veins dark red, apex acuminate, base oblique, margin serrulate to serrate, pilose on both surfaces with multicellular, branched, dark red trichomes, 6–7 pairs of main lateral veins, densely pilose; smaller blade highly reduced and often caducous, petiole 0.3–0.7 mm long; linear to lanceolate, 0.6–1.2 × 0.1–0.3 cm, lateral veins 1–3 pairs per side. ***Inflorescence*** reduced to 1–3 axillary flowers in a cluster; peduncle absent; bracts 4.7–7.7 × 1.7–2.7 mm, purplish, lanceolate, apex acuminate, base truncate, margin entire, pilose adaxially, glabrous abaxially. ***Flowers*** with erect pedicels, 2.5–2.7 mm long, purple, covered by multicellular, branched, dark red trichomes. ***Calyx*** uniformly dark red, 12.0–14.0 × 6.0–10.0 mm, persistent in fruit, mid and lateral veins not evident, calyx lobes 5, fused basally for 2.9–3.9 mm, lanceolate, apex attenuate, base truncate, margin laciniate, glabrous adaxially, densely pilose abaxially with dark red and branched multicellular trichomes. ***Corolla*** tubular, 3.0–3.8 cm long, tube erect relative to calyx, 8.4–9.6 mm wide at the middle, outer surface yellow and densely pilose with gold-branched trichomes, inner surface yellow and glabrous, corolla bases 4.0–6.0 mm wide, nectary chamber 4.0–7.0 × 3.0–5.0 mm, throat 5.0–6.2 mm diam., corolla lobes 5, 5.0–7.0 × 3.0–5.0 mm, yellow, straight, oblong, apex rounded, margin entire, densely pilose abaxially, with gold, multicellular trichomes, extending from the corolla lobes. ***Androecium*** of 4 didynamous stamens, filaments 18.0–23.0 mm long, adnate to the corolla tube for 8.0–10.0 mm, glabrous, staminode absent; anthers oblong, coherent, dehiscence by longitudinal slits, 1.4–1.6 × 2.2–2.6 mm. ***Gynoecium*** with a single dorsal trilobed nectary gland, ca. 3 mm long, glabrous, the middle lobe shorter than the lateral lobes; ovary superior, ca. 4.8 × 3.1 mm, dark yellow, ovate, pilose; style included, ca. 26 mm long, glabrous; stigma bilobed. ***Fruit*** an indehiscent berry, ca. 17.0 × 13.0 mm, conic, purple with red spots toward the apex, pilose with translucid trichomes.

#### Additional specimens examined.

**Colombia – Antioquia.** • Medellín: corregimiento San Antonio de Prado, Reserva asociada a cuencas, Astilleros, sendero principal cerca a Piedra Galeana, 6.2564109 N, 75.5937099 W, 2600 m, 10 Jan 2025 (fl.), *Y. Gallego-Franco et al. 795* (JAUM); • Municipio Medellín, corregimiento Palmitas, vereda La Volcana, trail following Quebrada Volcana, Cordillera Central, 6°20'34"N, 75°40'48"W, 2044 m, 11 May 2012 (fl.), *J.L. Clark, J. Anderson, L. Clavijo, Á. Idarraga & D. Suescún 12864* (COL, SEL); • Municipio Caldas, vereda La Clara, headwaters of Río Medellín, trail to Alto de San Miguel, Cordillera Central, 6°1'49"N, 75°36'45"W, 1990 m, 12 May 2012 (fl.), *J.L. Clark, J. Anderson, L. Clavijo & Á. Idarraga 12877* (COL, NY, SEL, US); • Municipio Medellín, municipio Envigado, Cordillera Central, camino La Catedral–Chorro de las Campanas, Valle La Miel, 6°7'29"N, 75°35'11"W, 2184 m, 15 May 2012 (fl.), *J.L. Clark, J. Anderson & T. Hinestroza 12290* (COL, SEL, US).

#### Phenology.

Plants were observed flowering in January, May, and August, and fruiting in August.

#### Etymology.

The specific epithet *rubropilosa* is derived from the Latin words *ruber* meaning “red” and *pilosus* meaning “hairy,” in reference to the conspicuous pilose indumentum of reddish trichomes that characterize the vegetative and reproductive structures of the species.

#### Distribution and preliminary IUCN red list assessment.

*Columnea rubropilosa* is endemic to Colombia and common throughout the Cordillera Central and eastern slopes of the Cordillera Occidental. It is located in the following protected areas: Reserva biológica El Centello managed by the Jardin Botánico Medellín and the Reserva Volcana Miserenga. There are more than 30 observations of *Columnea rubropilosa* on iNaturalist and most of these observations were posted during the last four years. It is especially abundant in the Cordillera Central south of Medellín where it appears to grow in shaded areas of secondary forest. Collections and observations from iNaturalist were used to calculate the AOO and EOO. Its extent of occurrence (EOO) is estimated at 26,975.116 km^2^, and its area of occupancy (AOO) is estimated at 96 km^2^. Given that it grows in shaded areas of secondary forest and its relative abundance from recent observations on iNaturalist, *Columnea rubropilosa* is preliminary assessed as Least Concern (LC).

#### Comments.

**Figure 3.**
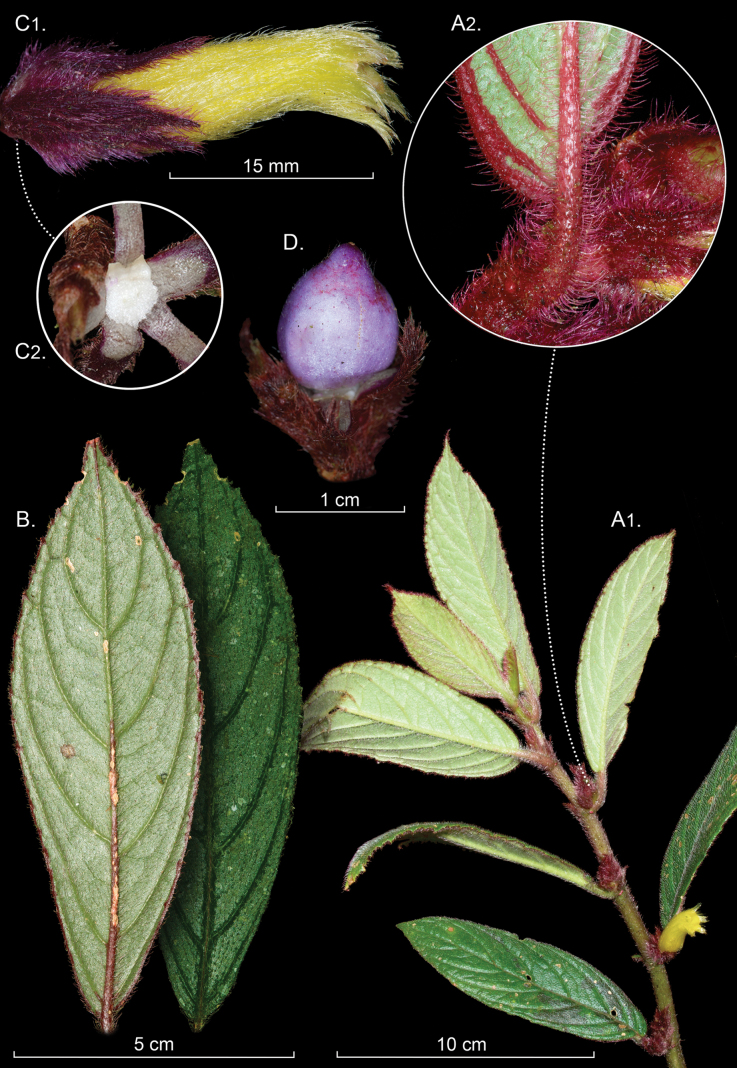
*
Columnea rubropilosa* J.L.Clark & Clavijo. A. Habit with inset featuring densely pilose indumentum; B. Abaxial and adaxial leaf surfaces; C. Lateral view of flower with inset featuring bilobed nectary gland; D. Fruit (A–D. from *J.L. Clark et al. 19325*). Photos by John L. Clark.

**Figure 4.**
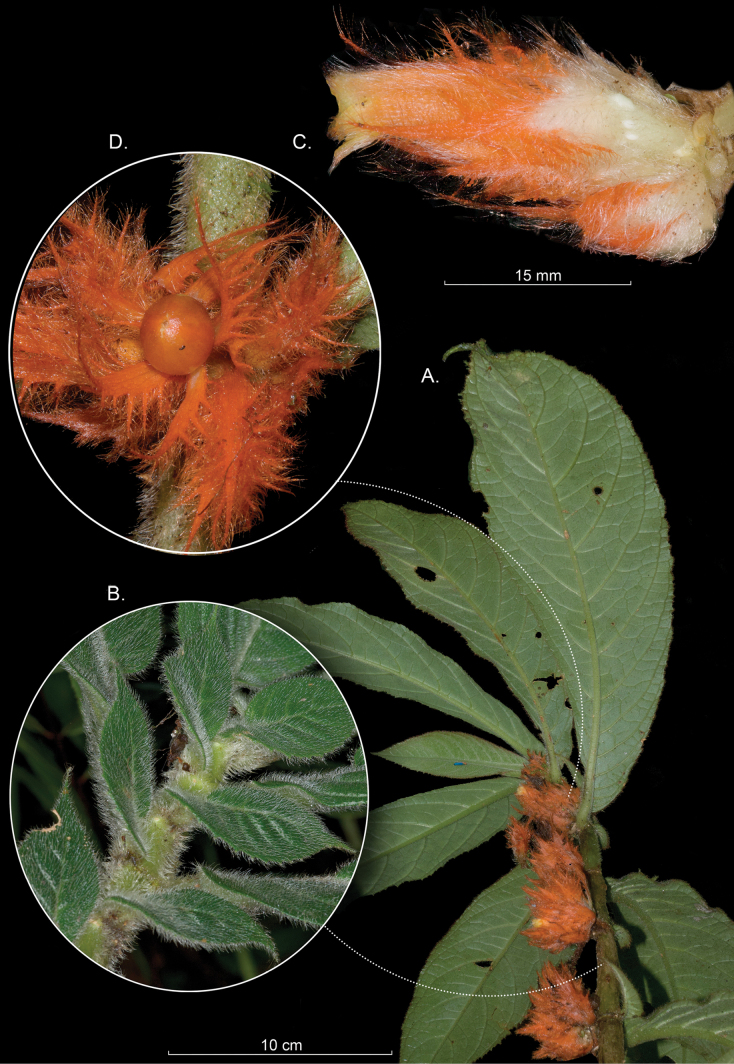
*
Columnea purpurata* Hanst. A. Habit; B. Adaxial surface of shoot featuring anisophyllous leaves; C. Lateral view of flower; D. Fruit (A–D. from *J.L. Clark et al. 12671*). Photos by John L. Clark.

*Columnea rubropilosa* is frequently observed on iNaturalist but identified as *Columnea purpurata*. Both species have conspicuous flowers, but with highly reduced floral bracts. In *C. rubropilosa*, the opposite leaves are anisophyllous (Fig. 3A), with the smaller leaf highly reduced and often caducous, resulting in phyllotaxy that appears alternate. In contrast, although the smaller leaf in *C. purpurata* is also reduced, it remains conspicuous, and the opposite leaf pairs are more readily apparent. The leaf blades of *C. rubropilosa* are stiff and nearly sclerophyllous, in contrast to the chartaceous leaves of *C. purpurata*. The calyx margins of *C. purpurata* are deeply fimbriate (Fig. 4D), whereas those of *C. rubropilosa* (Fig. 3D) are laciniate. Fruit color also differs: the berries in *C. rubropilosa* are purple (Fig. 3D) and orange in *C. purpurata* (Fig. 4D).

*Columnea rubropilosa* is one of the few known species of *Columnea* with a purple berry (Fig. 3D). The only other known species of *Columnea* with a purple berry is *Columnea conopurpurea* J.L. Clark, Y Rambos-Arias & J.L. Peña, which differs by an elongate-shaped berry. In contrast, the purple berry of *C. rubropilosa* is conic (Fig. 3D).

## Supplementary Material

XML Treatment for Columnea combeimae

XML Treatment for Columnea rubropilosa

## ﻿Additional information

### Use of AI

No use of AI was reported.

### Author ORCIDs

John L. Clark https://orcid.org/0000-0002-1414-6380

David Lozano-Cifuentes https://orcid.org/0000-0003-0353-4318

Jorge Ríos-Cervera https://orcid.org/0009-0009-1859-7355

Laura Clavijo https://orcid.org/0000-0002-3009-9158

Sofía Ballesteros https://orcid.org/0009-0005-1007-7735

### Data availability

All of the data that support the findings of this study are available in the main text.
